# Reliability of a Method to Quantify the Cortical Thickness of the Posterolateral Vertebral Arch on MRI

**DOI:** 10.1007/s43465-025-01504-4

**Published:** 2025-09-10

**Authors:** Richard Saw, Kevin Sims, Anna Saw, Jeremy Witchalls, David Connell, Alex Kountouris, Gordon Waddington, Greg Lovell, John Orchard

**Affiliations:** 1 Australian Institute of Sport, Leverrier St, Bruce, ACT Australia; 2 Research Institute for Sport and Exercise, University of Canberra, Bruce, ACT Australia; 3 School of Health and Rehabilitation Sciences, University of Queensland, St Lucia, QLD Australia; 4 Cricket Australia, Melbourne, VIC Australia; 5 Imaging @ Olympic Park, Melbourne, VIC Australia; 6 School of Public Health, University of Sydney, Sydney, NSW Australia

**Keywords:** Pars, Spondylolysis, Lumbar spine, Stress fracture, Bone stress

## Abstract

**Background:**

Lumbar stress fractures are very common lesions in young athletes participating in sport and are generally now diagnosed on MRI scanning. An ability to assess cortical thickness would help understand risk for future injury. This paper aims to establish a method for quantifying cortical thickness in the posterolateral vertebral arch of the lumbar spine on MRI scans.

**Methods:**

Methods for measuring the cortical thickness of the pars, pedicle, and lamina of the lumbar vertebra were developed from existing methods in other bones and expert opinion. MRI images of elite cricket pace bowlers were retrospectively reviewed in a development phase (*n* = 13 MRI, 30 measurement sites per MRI) and a reliability testing phase (inter-rater reliability *n* = 33 MRI, intra-rater reliability *n*-12 MRI).

**Results:**

Intra-rater reliability was overall excellent (intra-class correlation coefficient (ICC) 0.740–0.992). Inter-rater reliability ranged from fair to excellent (ICC 0.515–0.954), with lower reliability for the total pedicle cortex. Lower reliability was attributable to challenges of identifying the pedicle cortex and the more oblique orientation of the L5 vertebrae as the lumbar spine becomes more lordotic.

**Conclusion:**

This paper establishes a new manual method to quantify cortical thickness in the posterolateral vertebral arch of the lumbar spine on MRI scans. This work supports future advancements by providing a reference standard for potential automation using artificial intelligence and to understand how cortical thickness may be related to adaptation and injury in athletes at high risk of lumbar bone stress injury.

## Introduction

Lumbar bone stress injuries and chronic non-united defects (collectively referred to as “spondylolysis”) pose a considerable burden to athletic populations [1]. Lumbar bone stress injuries typically occur at the posterolateral vertebral arch and are associated with prolonged healing times, a relatively high incidence of non-union, and the potential for chronic low back pain, spondylolisthesis and degenerative disc disease as secondary complications from non-union [2–4]. Therefore, there is value in identifying individuals at higher risk of developing a lumbar bone stress injury to allow for intervention to reduce the injury burden. Bone architecture of the posterolateral vertebral arch is one factor which may provide insight into injury risk.

Bone architecture has innate qualities in certain groups and may also feature adaptations to sport-specific movements. For example, the bone mineral density of athletic populations is generally greater than their age-matched peers [5, 6] and may exhibit site-specific adaptations specific to athletic movement patterns [5, 7]. Sport-specific adaptations to bone require time for adequate remodelling and repair, potentially exposing the bone to an increased risk of stress lesion or fracture if loading continues before the bone adaptation can be completed [5, 8]. However, bone architecture is more complex than bone mineral density measurements can portray.

Studies in athletic cohorts point to thinner cortical thickness as a potential risk factor for bone stress injury. For example, female distance runners with a history of lower limb stress fracture(s) had lower tibial cortical bone area despite similar total bone area and diameter compared to uninjured peers [8], and female military recruits with a history of lower limb stress fracture(s) had thinner cortical thickness despite similar total bone diameter compared to uninjured peers [9]. Conversely, affected male military recruits had similar cortices but narrower total bone diameter [9]. A group of female and male triathletes with tibial bone stress had thinner anterior cortices and thicker medial and lateral cortices, although medial cortical thickness varied along the length of the tibia [10]. To date, cortical thickness of the posterolateral lumbar vertebrae has not been definitively associated with bone stress injury.

Cortical bone bears high axial loads in long bones and vertebrae [11–13]. The mechanical compressive and tensile strength of cortical bone is influenced by a number of architectural factors [11]. It has been suggested that as cortical bone has poorer tensile strength compared to compressive strength, repeated tensile loading leads to microcracks that are more prone to progressing under further tensile loads [11]. Therefore, cortical bone in the posterolateral lumbar vertebrae may be more susceptible to bone stress injury from tensile loading associated with the lumbar hyperextension and rotational movements of cricket pace bowlers, artistic and rhythmic gymnasts, soccer players, and javelin throwers [14–17]. Cadaveric modelling has been used to demonstrate that a lower proportion of cortical to trabecular bone in the posterolateral vertebral arch predisposed this area of bone to fracturing at a smaller number of repeated loadings [18].

To date, there is no established method for quantifying the cortical thickness of the posterolateral vertebral arch. Cortical thickness has been measured on magnetic resonance imaging (MRI) for the femoral neck [19], femoral shaft [20], tibia [10, 21], radius, ulna, and mandible [22]. The methods used to calculate cortical thickness in these studies include manual measurement on a 2D image, usually on T1 type sequences [21]. Other studies use computer-based algorithms or methods to automatically calculate the cortical thickness [23]. Advancements in automation through convolutional neural networks (CNN) or artificial intelligence (AI) computerised methods have the potential to improve the speed and reliability of measurement [24, 25]. However, to establish an AI-generated tool, a manual method of measurement must first be developed to guide the AI software [25]. Therefore, the purpose of this paper was to establish a method for quantifying cortical thickness in the posterolateral vertebral arch of the lumbar spine, and assess the reliability of this method.

## Materials and Methods

### Study Design

This retrospective review of stored images was approved through the University of Canberra—HREC—Project ID 9157 (transferred from Monash University HREC—Project ID 23024), which waived the need for informed consent. Images consisted of lumbar spine MRI of elite cricket pace bowlers (male and female) which had been completed for one of the following reasons: screening (asymptomatic), diagnostic (symptomatic), or assessing healing of lumbar bone stress injury.

Only MRI with volumetric interpolated breath-hold examination (VIBE) or equivalent sequences were included in this study. MRI of the lumbar spine in elite cricket pace bowlers are much more readily available than CT (which has some advantages in measuring cortical thickness) as MRI is widely used as the first-line imaging modality for lumbar bone stress injury [26–28].

### Methods

Lumbar bone stress injuries occur most commonly at the pars interarticularis, but can also occur at the pedicle, lamina, or into the transverse process or facet joints [29–31]. This has implications for where and how the posterolateral vertebral arch cortical thickness should be measured. Therefore, the cortical thickness of the posterolateral vertebral arch of the lumbar vertebrae was measured in three separate locations:

- Pars
- Pedicle
- Lamina

For each location, a novel method was established using manual measurements on the InteleViewer platform (Intelerad Medical Systems, Montreal, Canada). Reported methods for measuring cortical thickness on the femoral neck [19], femoral shaft [20], tibia [10, 21], and mandible [22] were referenced and adapted to be utilised for the posterolateral vertebral arch. The methods were developed through discussions and trials completed by a Musculoskeletal Radiologist, Sport and Exercise Medicine Physician, and a Sports Physiotherapist experienced in interpreting MRI in relation to lumbar bone stress injuries. The methods were refined using an iterative process and evaluated with inter-rater and intra-rater reliability. Reliability was calculated using a two-way random, absolute agreement intra-class correlation coefficient (ICC) with 95% confidence intervals (95% CI) (SPSS version 28, IBM, Armonk, NY, USA). Qualitative interpretation of ICC values above 0 (random agreement) is ICC < 0.40 poor, ICC = 0.40–0.59 fair, ICC = 0.60–0.74 good, and ICC = 0.75–1.00 excellent.[32] Correlations between measurement sites were assessed with linear regression.

The development phase of the method involved three raters independently measuring the cortical thickness of the pars, pedicle, and lamina of the left and right lumbar vertebrae L3 to L5 across 13 MRI (30 measurements per MRI). Measurement was limited to L3 to L5 as these levels are where the majority of lumbar bone stress injuries are reported [33]. The reliability of measurements across the three raters was overall poor. The raters then discussed discrepancies and refined the method to improve reliability. For example, the initial method involved using the apex of the superior facet of the vertebrae below as a reference point to measure the pars cortical thickness measure. This was changed to using an axial reconstructed view and InteleViewer 3D-cursor tool to cross-reference the pars location after the initial trial. When anatomical variations, current pathology or poor image quality impaired ease of measurement, the raters agreed to cross-reference the site in question with VIBE (or equivalent) images in another plane. If the rater remained not confident of accurately measuring the cortical thickness due to one of these factors, the measurement was marked as ‘unclear’ and excluded. Sites where a fracture was present (active or chronic) were excluded due to the inability to accurately measure the cortical thickness across a fracture.

The reliability testing phase of the method involved two raters (Sport and Exercise Medicine Physician and Physiotherapist) independently measuring 33 MRI (990 potential measurements) (inter-rater reliability). The method was trialled and assessed for reliability using existing MRI from elite Australian cricket pace bowlers. This cohort routinely undertake lumbar MRI as part of screening, diagnosis, and monitoring the healing of lumbar bone stress injuries. A randomised sample of MRIs from both male and female athletes was used. Intra-rater reliability was assessed by the Physician repeating measurements on 12 MRI (360 measurements) at least 7 days apart. The final methods used are detailed below.

**Fig. 1:**
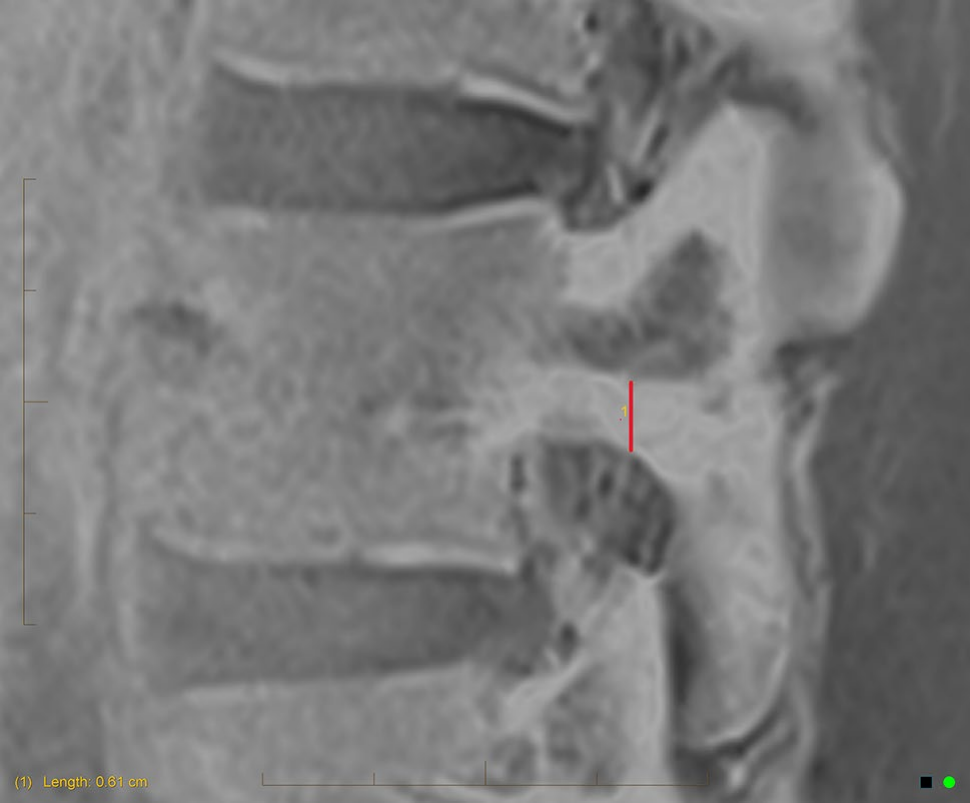
Pars cortical thickness measure

**Fig. 2:**
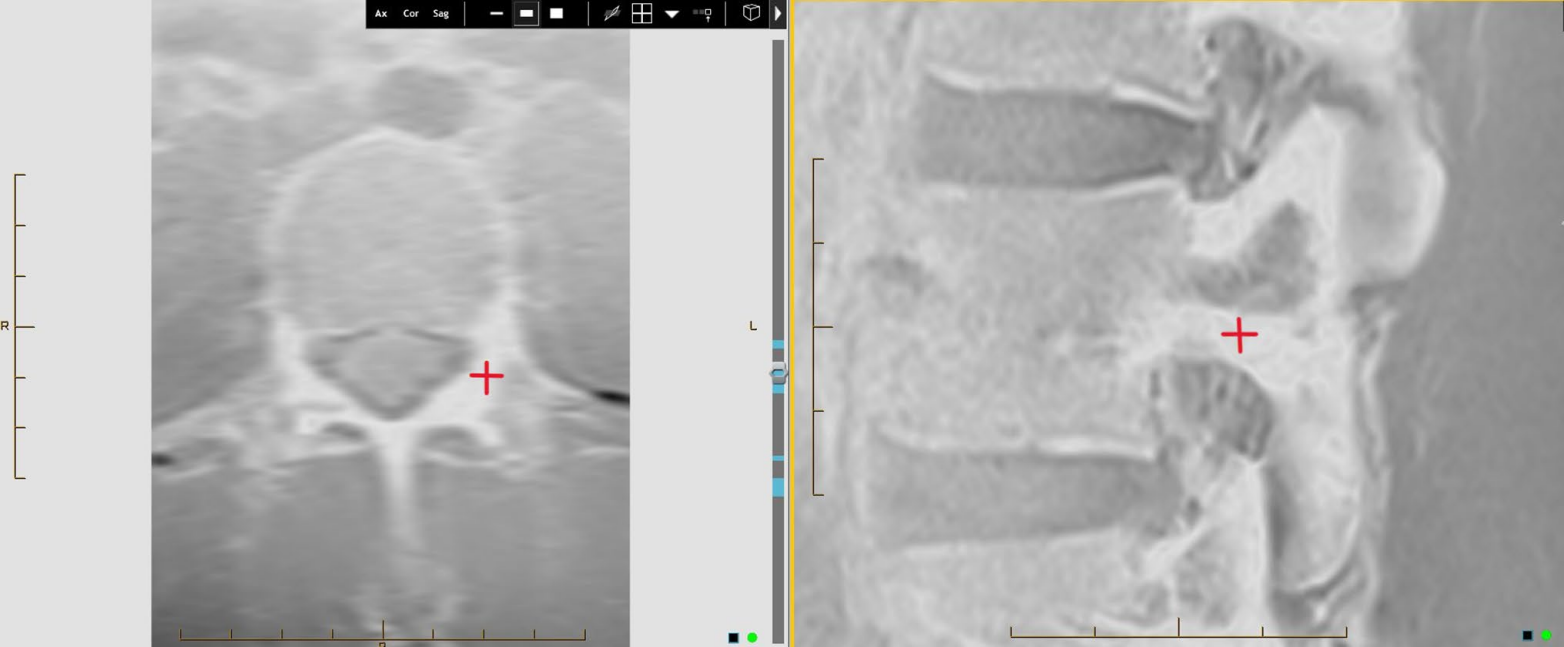
Cross-reference of the pars on axial image (left) to confirm the correct location to measure the pars cortical thickness on the sagittal image (right)

The pars cortical thickness was measured on sagittal slices of the VIBE (or equivalent modified T1) sequence. A vertical measurement was applied to the thickest cortical area (Fig. 1), that aligned with the pars on a 3D cross-reference of the equivalent axial slice (Fig. 2). Measurements were taken in centimetres to two decimal places.

**Fig. 3:**
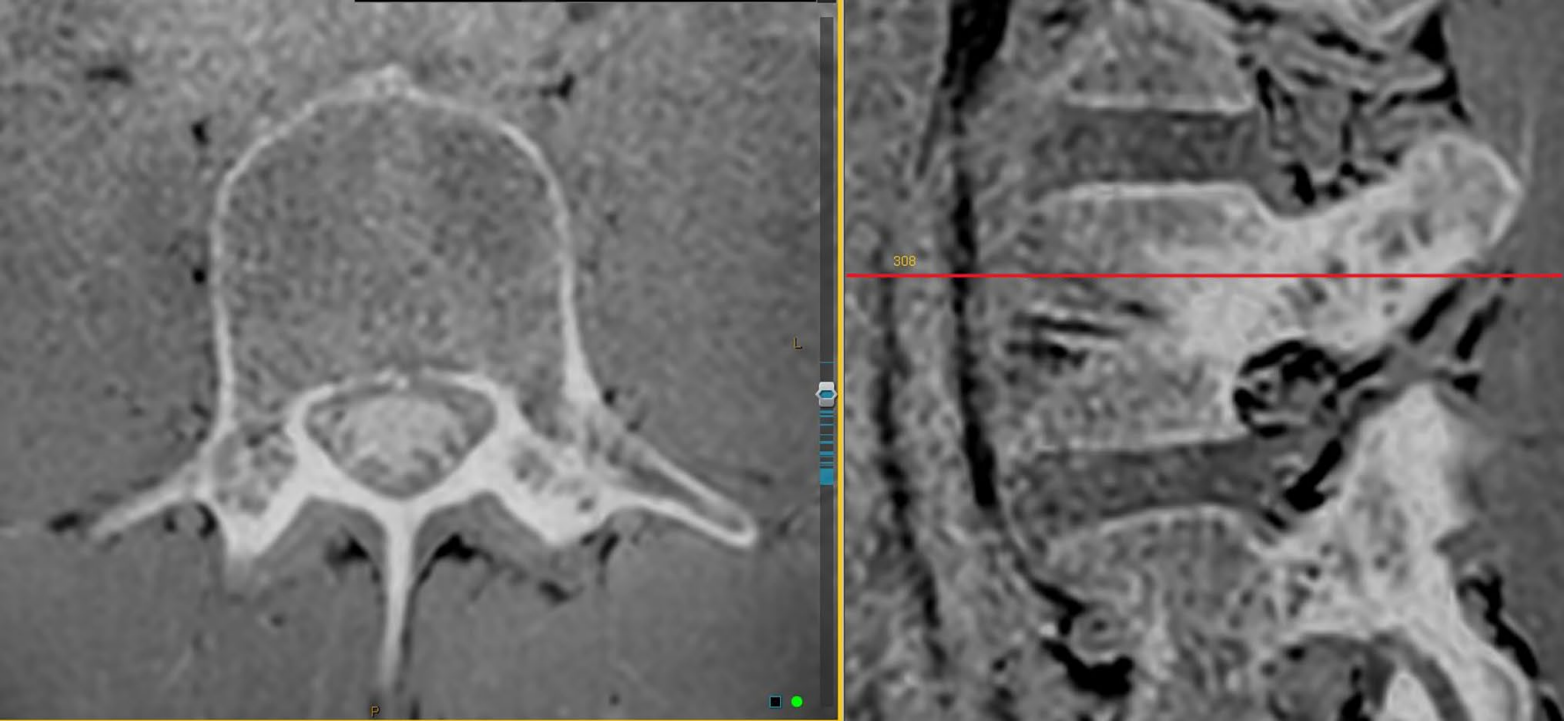
InteleViewer toggle reference line (red) used to ensure the axial image used for pedicle measurement is at the superior–inferior midpoint (right)

**Fig. 4:**
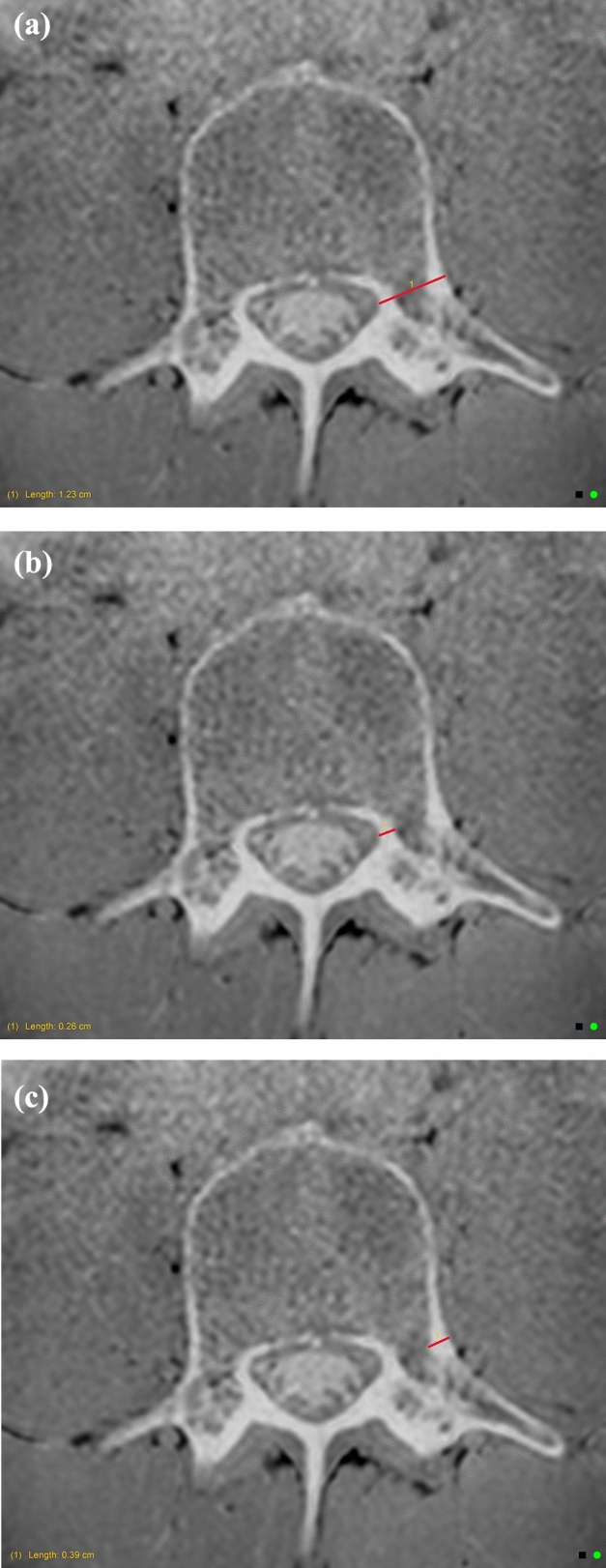
**a** Pedicle total width measurement. **b** Pedicle medial cortex measurement. **c** Pedicle lateral cortex measurement

The pedicle cortical thickness was measured on axial slices of the VIBE (or equivalent modified T1) sequence. The InteleViewer toggle reference line tool was used on the VIBE sagittal slice to locate the superior–inferior midpoint of the pedicle (Fig. 3). Three measurements were recorded: total pedicle width, medial cortex, and lateral cortex (Fig. 4a–c). The InteleViewer ruler tool was used to measure the shortest distance from the lateral apex of the vertebral foramen to the lateral edge of the pedicle. The medial and lateral cortex was measured in the same plane as the total pedicle width, by first shortening the ruler to measure the lateral cortex. To measure the medial cortex, the ruler was returned to the apex of the vertebral foramen to give the same total pedicle length measure, and then the ruler was shortened again from the lateral aspect to measure the medial cortex. The latter two measurements were combined to give a ‘total pedicle cortex’ measurement.

**Fig. 5:**
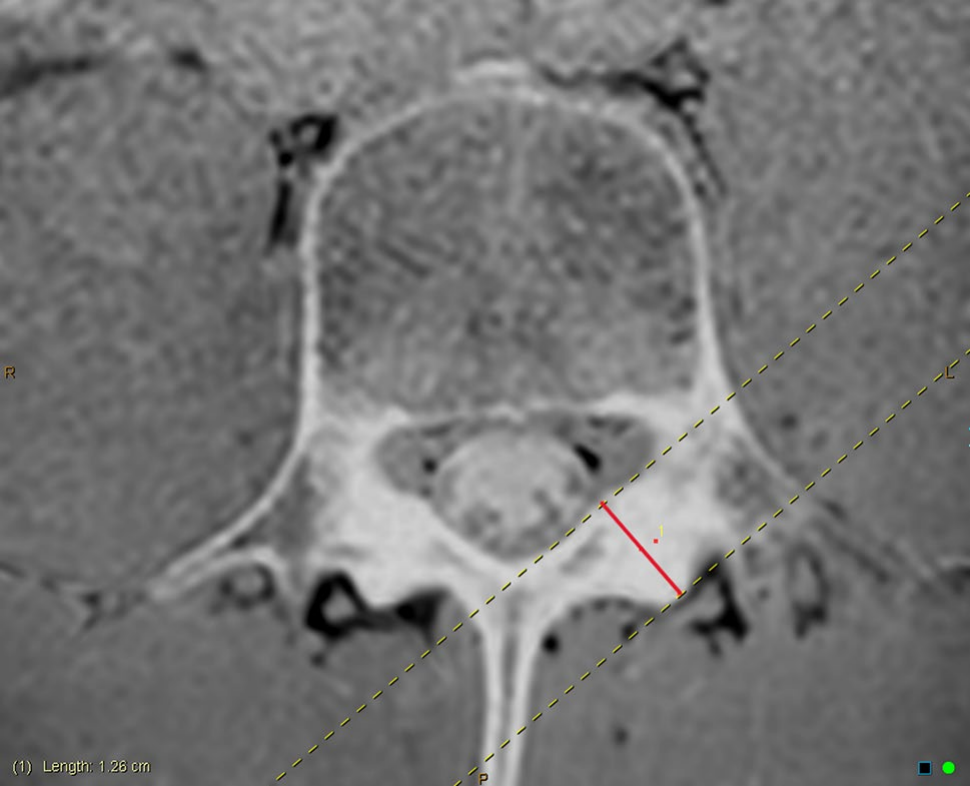
Lamina measurement

The lamina cortical thickness was measured on axial slices of the VIBE (or equivalent modified T1) sequence. Measurement was taken at the widest point of the lamina which did not extend into the facet joints, and measuring perpendicular to the vertebral foramen (Fig. 5). A total width measurement has been used for consistency, as bone was consistently visualised as being predominantly cortical bone in this region.

## Results

### Inter-rater Reliability

Across the 33 MRI (990 potential measurements), one or both of the raters marked 82 sites as ‘unclear’, and 7 had evidence of a fracture present. Eight MRI did not have a reformatted axial slice of the VIBE available, which precluded measurement of the pedicle and lamina (8 * 24 = 192 potential measurements). After excluding these measurements, a total of 709 pairs of data remained for analysis. Inter-rater reliability was fair-excellent across each measurement site and lumbar vertebrae as detailed in Table 1. The pedicle cortex as the sum of lateral and medial measurements had fair agreement across the three levels (ICC = 0.577), which was composed of lateral measurements with fair agreement (ICC = 0.498, 0.285–0.649) and medial measurements with excellent agreement (ICC = 0.854, 0.749–0.909). For each of the three vertebral levels, lateral pedicle measurements had poor-excellent agreement (L3 ICC = 0.469, 0.171–0.678; L4 ICC = 0.794, 0.569–0.895; L5 ICC = 0.330, 0.040–0.572) and medial pedicle measurements had fair-excellent agreement (L3 ICC = 0.541, 0.220–0.738; L4 ICC = 0.660, 0.447–0.798; L5 ICC = 0.930, 0.851–0.965).

**Table 1:** Intra-class correlation coefficients (95% confidence interval) for inter-rater reliability of test set of 33 MRI

Inter-rater (ICC, 95% CI)	Pars	Pedicle total	Pedicle cortex sum (lateral + medial)	Lamina
All levels (*n* = 161,143,142,126)	0.700 (0.572–0.787)	0.954 (0.936–0.983)	0.577 (0.313–0.732)	0.880 (0.819–0.919)
L3 (*n* = 60,49,49,46)	0.698 (0.542–0.808)	0.899 (0.828–0.942)	0.565 (0.183–0.769)	0.696 (0.508–0.820)
L4 (*n* = 50,49,48,47)	0.649 (0.387–0.801)	0.906 (0.838–0.946)	0.661 (0.368–0.817)	0.880 (0.790–0.932)
L5 (*n* = 51,45,45,33)	0.717 (0.506–0.839)	0.896 (0.812–0.942)	0.515 (0.175–0.726)	0.884 (0.755–0.944)

*n* reflects the actual number of measurements included in reliability analysis, including left and right at each region and level, excluding sites marked unsure or with a fracture

### Intra-rater Reliability

Across the 12 MRI (360 measurements), 268 pairs of data were available for analysis. Intra-rater agreement was overall excellent. Intra-rater reliability for each measurement site and lumbar level was excellent for all levels and measures except ‘pedicle cortex sum’ at L5 only, which at ICC 0.740 was narrowly below the excellent range (Table 2).

**Table 2:** Intra-class correlation coefficients (95% confidence interval) for intra-rater reliability of 12 MRI

Intra-rater (ICC, 95% CI)	Pars	Pedicle total	Pedicle cortex sum (lateral + medial)	Lamina
All levels (*n* = 67,67,24,67)	0.882 (0.815–0.926)	0.991 (0.986–0.995)	0.808 (0.606–0.912)	0.905 (0.793–0.951)
L3 (*n* = 24,24,24,24)	0.861 (0.705–0.938)	0.992 (0.982–0.997)	0.808 (0.606–0.912)	0.872 (0.604–0.951)
L4 (*n* = 22,23,23,24)	0.846 (0.655–0.934)	0.961 (0.912–0.983)	0.921 (0.824–0.966)	0.972 (0.807–0.992)
L5 (*n* = 21,20,21,19)	0.903 (0.779–0.959)	0.982 (0.955–0.993)	0.740 (0.461–0.886)	0.791 (0.535–0.914)

### Correlations

All iterations of pairs of measurement sites were weakly correlated, with the exception of a stronger correlation between pedicle total and lamina measures (Table 3).

**Table 3:**
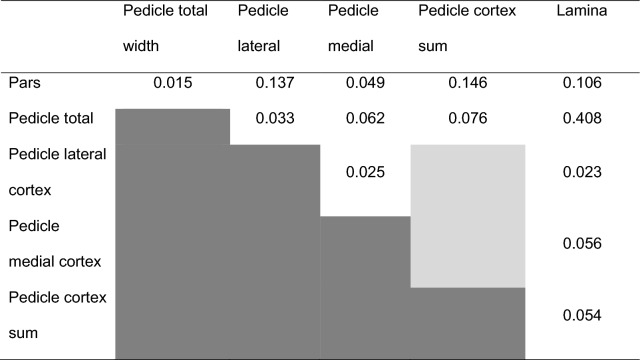
R-squared values for linear relationship between measurement sites of 33 MRI

## Discussion

Cortical thickness of the lumbar spine may be relevant to understand both adaptive and maladaptive features related to sport-specific movements and risk of bone stress injury.

This paper establishes a new manual method to quantify cortical thickness in the posterolateral vertebral arch of the lumbar spine. Using clear definitions and cross-referencing tools to identify precise measurement sites of the pars, pedicle, and lamina, the methods were shown to have overall excellent reliability.

The reliability of measurements is subject to error introduced by slight variation in image orientation and a degree of subjectivity in selecting the exact points of measurement. The reliability at L5 may have been affected by the more oblique orientation of the L5 vertebrae as the lumbar spine progresses into more lordosis. The pedicle cortex involved the sum of medial and lateral measurements, thus increasing the potential for error.

Reliability may have been improved with the use of computerised tomography (CT). However, measurements of other non-cortical lumbar spine geometry on MRI have been shown to correlate well with measurements on CT [34], and MRI is commonly the first-line option for diagnosing bone stress injuries and other non-bony pathology, without radiation [26]. Diagnosis of lumbar stress fractures correlates well with CT [35]. MRI may also be used for early lumbar bone stress injury detection [28] and to assess bone healing [27] in certain high-performance athlete populations. Therefore, MRI images of the lumbar spine in groups at risk of lumbar bone stress injury are more readily available compared to CT.

Practical limitations of the methods are that they are manual, time consuming, and require experience and understanding of the anatomy of the posterolateral vertebral arch, recognising factors that would impede accurate measurement (for example a current stress fracture), and skills using the InteleViewer platform. Artificial intelligence may address these limitations, using the manual methods described in this paper as a reference standard.

Establishing methods to measure the cortical thickness of the posterolateral vertebral arch lends to future research directions. This includes developing sport-specific normative data (for example cricket pace bowlers, stratified by factors such as gender and age). The influence of cortical thickness on lumbar bone stress injury risk can also be further investigated. This has potential implications for assessing lumbar bone stress injury risk and implementing injury prevention strategies for high-risk athletes. The weak correlation between all iterations of pairs of measurement sites suggests they are not clinically interchangeable, and the clinical utility of each measurement will need to be considered independently in future research.

In conclusion, this paper establishes a new manual method to quantify cortical thickness in the posterolateral vertebral arch of the lumbar spine with overall excellent reliability. This work will help understand how cortical thickness may be related to adaptation and injury in athletes at high risk of lumbar bone stress injury, and supports future advancements by providing a reference standard for potential automation using artificial intelligence.

## Data Availability

Not applicable.
